# High IL-10 mRNA levels in regional lymph nodes of colon cancer patients indicate poor prognosis

**DOI:** 10.3389/fimmu.2025.1589533

**Published:** 2025-09-23

**Authors:** Alaa Mohamed, Hager Ismail, Faten Zahran, Nabila Zein, Gudrun Lindmark, Marie-Louise Hammarström, Sten Hammarström, Basel Sitohy

**Affiliations:** ^1^ Department of Clinical Microbiology, Umea University, Umea, Sweden; ^2^ Department of Diagnostics and Intervention, Umea University, Umea, Sweden; ^3^ Department of Biochemistry, Faculty of Science, Zagazig University, Zagazig, Egypt; ^4^ Department of Clinical Pathology, Faculty of Veterinary Medicine, Zagazig University, Zagazig, Egypt; ^5^ Institution of Clinical Sciences, Lund University, Lund, Sweden

**Keywords:** colon cancer, regional lymph node, FoxP3, IL-10, LGR6, prognosis, qRT-PCR, immunohistochemistry

## Abstract

**Introduction:**

The prognostic value of determining mRNA levels of two markers for regulatory T cells, IL-10 and FoxP3, in lymph nodes (LNs) and primary tumors of colon cancer (CC) patients receiving curative surgery was investigated.

**Methods:**

mRNA levels were determined by real-time qRT-PCR in 370 LNs from 120 CC patients representing all four TNM stages, 66 primary tumors, 30 normal colon tissue samples and appropriate cell lines. Protein expression was analyzed by immunohistochemistry. Patients were followed for 12 years.

**Results:**

High levels of IL-10 mRNA in LNs were associated with poor prognosis with shorter mean survival time of 10 and 32 months (*p* = 0.001 and *p* = 0.004) at 5- and 12-year follow-up with hazard ratios of 12.4 and 6.3, respectively. No association between IL-10 level and prognosis was seen in the primary tumor. High levels of FoxP3 mRNA were associated with good prognosis, both in LNs and primary tumor. The difference in survival time was, however, small. Analysis of IL-10 mRNA in combination with LGR6 or CXCL17 mRNA in LNs generated patients with different risk of recurrence – low-, high- and very high risk. Immunohistochemistry identified IL-10 and FoxP3 positive cells located at the outer rim of tumor aggregates.

**Conclusion:**

Level determinations of IL-10 mRNA in LNs are useful for prediction of outcome for CC patients after curative surgery. Low levels indicate that the patients do not require further treatment, while IL-10 in combination with LGR6 or CXCL17 can be used to identify patients at very high risk of recurrence.

## 1 Introduction

Colon cancer (CC) is one of the most prevalent causes of cancer death worldwide. Although the incidence and death of CC are decreasing in several countries due to colonoscopy screening and improved treatment, CC still ranks third globally in morbidity and second in mortality (1). Surgery is the primary therapeutic option, often supplemented with other treatments including chemotherapy, anti-angiogenic therapy, and immunotherapy (2, 3).

The immune system is thought to play an important role in defending the individual from tumor development through effector cells of both adaptive and innate immunity. Different immune cells, including αβT-cells, γδT-cells, NK-cells, macrophages and dendritic cells participate in the defense. Tumor antigen directed CD8^+^T-cells with cytotoxic properties are implicated as particularly important effector cells in the anti-tumor defense. The immune system has many roles and includes cells with regulatory properties (Tregs) that are needed to maintain immune homeostasis, promote self-tolerance and prevent too excessive immune responses. Tregs express the transcription factor forkhead box protein P3 (FoxP3) and the cytokine interleukin-10 (IL-10). It is thought that tumor cells will defend themselves by manipulating the immune system either directly by expressing and releasing inhibitory factors and/or by over-activating regulatory T cells to release inhibitory factors such as the cytokine IL-10 (4–6).

IL-10 is a member of the class II cytokine family. The biologically active form is a soluble 36 kDa homodimer. To achieve a biological effect the IL-10 homodimer needs to interact with the heterotetrameric IL-10 receptor (IL-10R) on the responding cell. The receptor is composed of two ligand-binding subunits (IL-10RA) and two accessory-signal transducing IL-10RB subunits (7). Major cellular sources of IL-10 are T-cells including Tregs and TR1 cells (8). However, B-cells and monocytes/macrophages can also express IL-10 (8).

Of particular interest in relation to colorectal cancer (CRC) is that IL-10 plays a central role in normal intestinal homeostasis. Thus, IL-10 deficient mice spontaneously develop colitis while IL-10 deficient germ-free mice do not, indicating that IL-10 maintains tolerance to the commensal intestinal microflora (9). Genetic defects in the IL-10/IL-10R pathway in humans similarly lead to the development of severe early-onset colitis which can be cured by transplantation of stem cells producing the cytokine or cytokine receptor (10). These data point to a non-redundant role of IL-10. The role of IL-10 in the immune system is versatile - not only can IL-10 inhibit the synthesis of a variety of cytokines, both inflammatory and growth promoting, it can promote secretion of anti-inflammatory factors and regulate differentiation and proliferation of several types of immune cells. As a result of these opposing functions of IL-10, it may promote or inhibit tumor formation and progression depending on tumor type and stage in tumor development (11–15).

The transcription factor FoxP3 is identified as an essential factor for the function of Tregs and an obligatory marker of CD4^+^CD25^+^Tregs (16). Immune cell suppression by Tregs is achieved by production and release of cytokines, including IL-10 and transforming growth factor-β (TGF-β) (17). The number of Tregs in primary CRC tumor tissue is significantly higher than in normal colon tissue and most interestingly this difference is also seen in the draining lymph nodes (LNs) of the patients. Thus, metastatic LNs harbor significantly higher numbers of Tregs than non-metastatic LNs (18). As reviewed by D. Mougiakakos (19), based on ten independent studies, it is still an open question whether high numbers of Tregs, as defined by immunohistochemistry using antibodies against FoxP3 in analysis of the primary tumor, is prognostic in CRC. A more recent study (20) showed that patients with high FoxP3^+^ Treg density had significantly improved 5-year survival rate compared with those with low density. The Tregs were found in the invasive margin of the primary tumor.

It has become increasingly clear that neither single markers nor combinations thereof can define all and only Tregs. To date, only the functional capacity to inhibit immune responses defines a Treg and distinguishes Tregs from inflammatory T cells (Teffs) in humans (21).

In this study, we focus on mRNA analysis to investigate levels of IL-10 and FoxP3 in LNs of CC patients of all four TNM stages correlating levels to relapse in cancer up to 12 years after surgery. For comparative purposes the mRNA levels of the two biomarkers in the primary tumor were also investigated. Two new highly specific qRT-PCR assays with RNA copy standards for determining absolute expression levels were used. A limited number of tumors and LNs were also studied by immunohistochemistry. The most important finding was that high IL-10 mRNA levels in LNs was predictive of bad prognosis.

## 2 Materials and methods

### 2.1 The objectives of the study

The primary objective is to determine the IL-10 and FoxP3 mRNA levels by real-time qRT-PCR in 370 LNs from 120 CC patients representing all four TNM stages, 66 primary tumors, 30 control colon tissue samples and appropriate cell lines.

The secondary objective is to assess the prognostic value of IL-10 and FoxP3 mRNA levels in CC patients using Kaplan-Meier survival model and Cox regression analyses and examining the combined prognostic value of IL-10 with LGR6 and CXCL17 mRNAs.

### 2.2 Patients and study design

One hundred and twenty patients in whom locally radical tumor resection for CC was carried out were included on a continuous basis at two Swedish sites, the Norrland University Hospital in Umea and the Helsingborg Hospital in Helsingborg from November 2001 until February 2008. Inclusion criteria were primary surgery with intention to cure, willingness to participate in the study and no other cancer, except skin cancer excluding melanoma. LNs were collected from the resected specimen and bisected by the surgeon in the operating room. One half was formalin-fixed, embedded in an individual paraffin block and used in routine histopathology examination for pN classification. The other half was snap-frozen as fresh tissue and stored at -70 °C until RNA extraction. A total of 370 LNs, on average 3 per patient (range 1 - 13), were collected. Based on histopathology, 20 LNs were judged metastatic [H&E(+)], and 350 LNs were judged non-metastatic [H&E (–)]. A fresh tissue specimen of the primary tumor was also collected from 66 of the patients. These samples were treated in the same way as LNs and stored at -70 °C until RNA extraction. Tumor stage as judged by histopathology was pT2 (n=13), pT3 (n=42), and pT4 (n=11). Control LNs (n = 77) were collected from 13 patients (10 males and 3 females) with a median age of 23 years (range: 9-32), undergoing surgery for lipoma (n=1), Crohn’s disease (n=1), and ulcerative colitis (n=11). Normal colon tissue specimens for mRNA analysis were collected from the distal resection margin of 30 CC patients.

Immunohistochemistry was performed on 13 LNs, 10 primary tumor samples and 9 normal colon tissue samples. Seven of the LNs were H&E(+). These were from five stage III patients. Six LNs were H&E (–). These were from one patient in stage I, two in stage II, and two in stage III. Tumor stage of the primary tumors was: pT2 (n=1), pT3 (n=6), and pT4 (n=3).

Clinical features of the CC patients are shown in **Table 1**. None of the patients received chemotherapy before surgery. Outcome measure in survival analysis was relapse of cancer or cancer-specific death at 5-year and 12-year follow-up. No patients were lost at follow-up.

**Table 1:** Clinical features of colon cancer (CC) patients who donated primary tumor tissue, normal colon tissue and lymph nodes for mRNA and immunohistochemistry (IHC) analyses.

Type of analysis	Clinical features of colon cancer (CC) patients		Type of analyzed tissue		
			Primary tumor	Normal colon ^&^	Lymph nodes ^#^
mRNA	N *		66	30	120
	Gender	Male	30	17	54
		Female	36	13	66
	Age (years)	Median	74	72	74
		Range	(42-89)	(57-85)	(43-89)
	TNM stage	I	14	7	23
		II	30	17	52
		III	17	4	36
		IV	5	2	9
IHC	N *		10	9	10
	Gender	Male	5	4	2
		Female	5	5	8
	Age (years)	Median	72	70	80
		Range	(60-84)	(41-83)	(71-91)
	TNM stage	I	1	1	1
		II	3	3	2
		III	4	2	7
		IV	2	3	–

* N: total number of CC patients who had donated tissue specimens of primary tumor, normal colon, and lymph nodes, respectively.

^&^Normal colon tissue: specimens gathered from the resection margins of primary tumors, distant from macroscopically detectable lesions.

^#^Lymph nodes of CC patients for mRNA analysis were retrieved from 120 CC patients. A total of 370 lymph nodes were gathered, of these, were 70 from 23 patients in stage I, 186 from 52 patients in stage II, 85 from 36 patients in stage III, and 29 from 9 patients in stage IV.

Lymph nodes of CC patients for IHC were retrieved from 10 CC patients. A total of 13 lymph nodes were analyzed, seven of these were metastatic, retrieved from five patients in stage III, and six were non-metastatic, retrieved from one patient in stage I, two patients in stage II, and two patients in stage III.

### 2.3 Cell lines

Total-RNA from 5 human CC cell lines (Caco2, T84, HCT8, HT29, LS174T), 1 human T cell line (Jurkat), 2 human B cell lines (CNB6, KR4), 1 human monocyte cell line (U937), 1 endothelial cell line (HUVEC), and primary foreskin fibroblasts (FSU) were from previous studies (22–25).

### 2.4 Real-time quantitative reverse transcriptase-polymerase chain reaction

qRT-PCR assays were constructed for absolute quantification of IL-10 and FoxP3 mRNAs, employing primers positioned in different exons, a reporter dye-labeled probe hybridizing over the exon boundary in the amplicon and specific RNA copy standards for the quantification. The IL-10 mRNA assay detects two transcript variants, NM_000572.3 and NM_001382624.1. The primers and probe sequences for IL-10 mRNA were forward primer 5'-GGAGAACCTGAAGACCCTCA-3', reverse primer 5'-TGCTCTTGTTTTCACAGGGA-3', and probe 5'-AGGCTACGGCGCTGTCATCGATTTC-3'. The FoxP3 mRNA assay detects two transcript variants, NM_014009.4 and NM_001114377.2. The primers and probe sequences for FoxP3 mRNA were forward primer 5'-GCACCTTCCCAAATCCCAGT-3', reverse primer 5'-GGCCACTTGCAGACACCAT-3' and probe 5'-CAGGAAGGACAGCACCCTTTCGGC-3'. FAM was the reporter dye, and NFQ-MGB was the quencher dye. The amplicon size was 70 bases for IL-10 and 107 bases for FoxP3. The qRT-PCR profile was 60 °C for 5 min and 95 °C for 1 min, followed by 45 cycles of 95 °C for 15 s and 60 °C for 1 min. RNA oligonucleotides with sequences identical to those in the areas amplified in the respective qRT-PCR assay were custom synthesized at Dharmacon (Lafayette, CO, USA) and used as RNA copy standards. Serial dilutions of the RNA copy standards at concentrations from 10^3^ to 10^8^ copies/μL were included in each qRT-PCR run. Concentrations of mRNAs in unknown samples were set from the standard curve and expressed as copies of mRNA/μL. The concentration of 18S rRNA was determined in each sample using a commercial real-time qRT-PCR assay (Applied Biosystems) and expressed as arbitrary units from a standard curve of serial dilutions of preparation of total RNA from human peripheral blood mononuclear cells. One unit was defined as the amount of 18S rRNA in 10 pg RNA (26). FoxP3 and IL-10 mRNA levels were expressed as copies/18S rRNA unit. qRT-PCR assays for carcinoembryonic antigen (CEA), C-X-C motif chemokine ligand 17 (CXCL17), and leucine rich repeat containing G protein-coupled receptor 6 (LGR6) mRNAs have previously been described (27–29).

### 2.5 Immunohistochemistry reagents

Mouse monoclonal antibodies (mAbs) against human IL-10 (IgG, Cat. No. 604-950, AbboMax, Inc. USA), human FoxP3 (IgG, eBioscience, Cat. No. 14-4777-82, ThermoFisher, USA), and CEA (IgG1, clone II-7, Dako, Glostrup, Denmark) were used. Mouse IgG ready-to-use (Dako), served as a negative control. The anti-mouse IgG ImmPRESS enhancement kit was used as a secondary reagent (Vector Laboratories, Burlingame, CA, USA), and the substrate used was 3,3′-diaminobenzidine (DAB; Vector Laboratories).

### 2.6 Immunohistochemistry procedure

Fresh tissue samples were rinsed with cold phosphate-buffered saline (PBS), snap-frozen in isopentane pre-cooled in liquid nitrogen, and stored at -70 °C. Frozen tissue was cut into 4–6 μm thick sections with a cryo-microtome (MICROM HM505E, Thermo Fisher, Waltham, MA, USA). As described previously (30, 31), the sections were fixed with 4% paraformaldehyde for 15 minutes, then air dried, rehydrated in PBS, and immersed in PBS containing 0.03% H_2_O_2_ and 2 mM NaN_3_ at 37 °C to quench endogenous peroxidase activity. Subsequently, the sections were incubated with 0.2% bovine serum albumin in PBS, which was followed by ImmPRESS ready-to-use horse-blocking serum (Vector Laboratories) at room temperature to block non-specific binding sites. Subsequently, the sections were incubated with primary antibodies, followed by incubation with ImmPRESS anti-mouse IgG. Bound peroxidase was revealed by incubation with 0.05% DAB and 0.03% H_2_O_2_ in 0.05 M Tris buffer (pH 7.6) at room temperature and was counterstained with methyl green. Anti-CEA mAb and mouse IgG instead of primary antibody served as positive and negative controls, respectively.

### 2.7 Statistical analysis

The statistical significance of differences in mRNA levels between primary CC tumors and normal colon tissues was calculated using two-tailed Mann-Whitney rank sum test. Kruskal-Wallis one-way analysis of variance (ANOVA) test, followed by Dunn's multiple comparison *post hoc* test, was employed to analyze statistical significance of differences in mRNA levels in LNs from various TNM stages, as well as in H&E(+) versus H&E(–) and control LNs, and LNs with different CEA levels. Non-parametric Spearman correlation coefficient test was used to evaluate correlations between mRNA levels of different biomarkers in primary tumors and LNs. The software utilized for statistical calculations was GraphPad Prism 9 (GraphPad Software, San Diego, CA, USA). Patients were grouped into those with high and low IL-10 and FoxP3 levels, respectively, using the median level across all analyzed samples of the respective mRNA as cut-off as a starting point. If no difference in disease-free survival was seen between the high and the low group, other cut-offs based on for instance sample distribution were investigated and used if a significant difference between the groups was found. The SPSS software (IBM Corporation, Armonk, NY, USA) was used for statistical analyses of differences between patient groups in disease-free survival time and analyses of risk of recurrent disease after surgery, according to the Kaplan-Meier survival model in combination with the log-rank test and univariate Cox regression analysis, respectively. A *p*-value ≤0.05 was considered statistically significant.

## 3 Results

### 3.1 Analysis of primary CC tumor and cell lines

**Figure 1:**
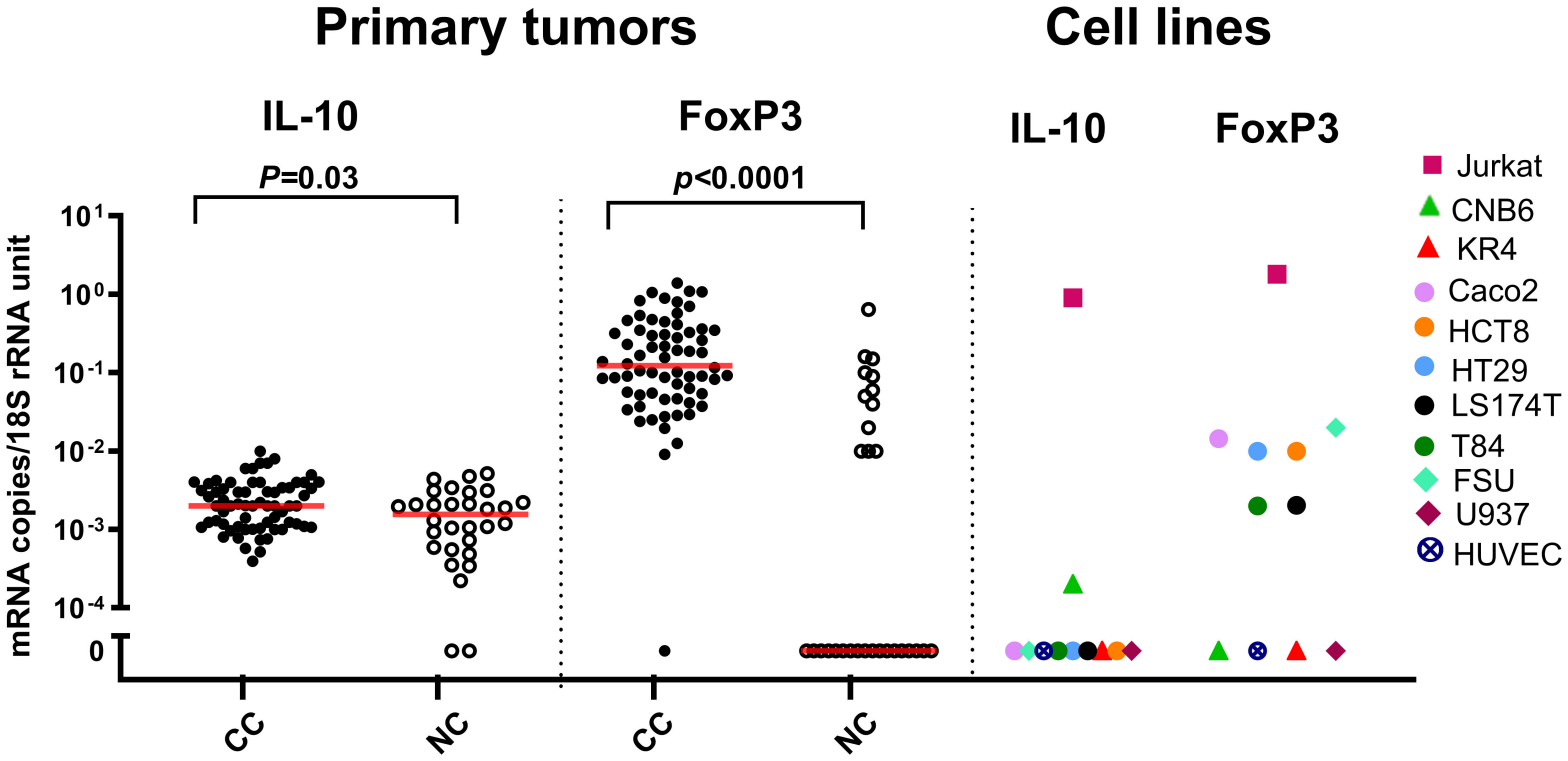
IL-10 and FoxP3 mRNA levels in primary tumor and cell lines. Levels of IL-10 and FoxP3 mRNAs in primary tumor tissue of colon cancer patients (CC), resected normal colon tissue (NC), and in a panel of colon cancer cell lines (Caco2, HCT8, HT29, LS174T, T84), a T-cell line (Jurkat), two B-cell lines (CNB6 and KR4), a monocyte cell line (U937), primary foreskin fibroblast cells (FSU), and an endothelial cell line (HUVEC). mRNA levels are quantified as mRNA copies/18S rRNA unit. Red horizontal bars indicate the median values. *p*-values were determined using two-tailed Mann-Whitney rank sum test.

The mRNA expression levels of IL-10 and FoxP3 in primary CC tumor, normal colon tissue, CC cell lines and immune cell lines are shown in **Figure 1**. IL-10 mRNA levels were slightly higher in primary tumors than in normal colon tissue (median 0.002 and 0.0015 mRNA copies/18S rRNA unit, respectively). For FoxP3 mRNA the difference in levels between primary tumor and normal colon tissue was larger (median 0.13 and 0.001 mRNA copies per 18S rRNA unit, respectively), mainly due to the fact that a major fraction of the normal colon samples did not express FoxP3 mRNA at all. Most interestingly, none of the five CC cell lines expressed IL-10 while IL-10 was expressed at high levels in the T-cell line Jurkat and at low levels in the B-cell line CNB6. FoxP3 mRNA was expressed at high levels in the T-cell line and at low levels in all five CC cell lines and the fibroblast cell line.

**Figure 2:**
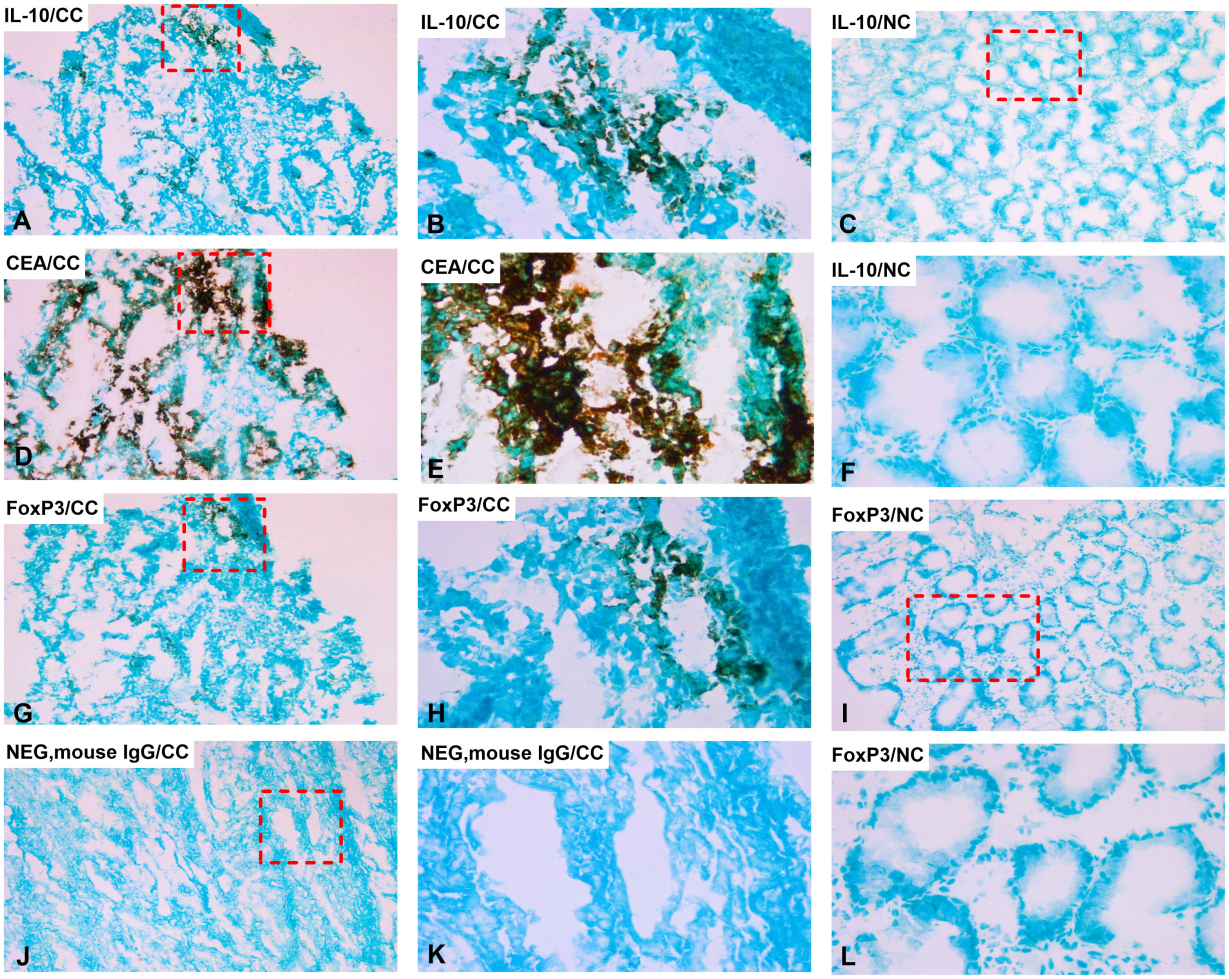
Distribution of IL-10 and FoxP3 positive cells in primary CC tumor and normal colon tissue. Immunoperoxidase stained tissue sections of a primary tumor tissue of colon cancer patients in **(A, B, D, E, G, H, J, K)** and normal colon tissue in **(C, F, I, L)**. **(A)** primary tumor tissue stained with anti-IL-10 mAb in a consecutive section of **(D)**, original magnification 100×. **(B)** higher magnification of the area indicated by a hatched box in **(A)**, 400×. **(C)** normal colon tissue stained with anti-IL-10 mAb, original magnification 100×. **(D)** primary tumor tissue stained with anti-CEA mAb, original magnification 100×. **(E)** higher magnification of the area indicated by a hatched box in **(D)**, 400×. **(F)** higher magnification of the area indicated by a hatched box in **(C)**, 400×. **(G)** primary tumor tissue stained with anti-FoxP3 mAb in a consecutive section of **(D)**, original magnification 100×. **(H)** higher magnification of the area indicated by a hatched box in **(G)**, 400×. **(I)** normal colon tissue stained with anti-FoxP3 mAb, original magnification 100×. **(J)** negative control (mouse IgG) of a primary tumor tissue, original magnification 100×. **(K)** higher magnification of the area indicated by a hatched box in **(J)**, 400×. **(L)** higher magnification of the area indicated by a hatched box in **(I)**, 400×. **(A, D, G)** are consecutive sections.

IL-10 and FoxP3 protein expression were analyzed by immunohistochemistry using the consecutive section staining technique. To identify the tumor cells in the primary CC tumor we used staining with anti-CEA mAb. As can be seen in **Figures 2D, E** aggregates of tumor cells are clearly identified in the tissue section. Both anti-IL-10 and anti-FoxP3 (**Figures 2A, B, G, H**) stained cellular structures in the proximity of some of the tumor cell aggregates while no staining was seen in the normal colon tissue (**Figures 2C, F, I, L**). Taken together, these results indicate that IL-10 and probably also FoxP3 are expressed by cells in the tumor cell environment.

**Figure 3:**
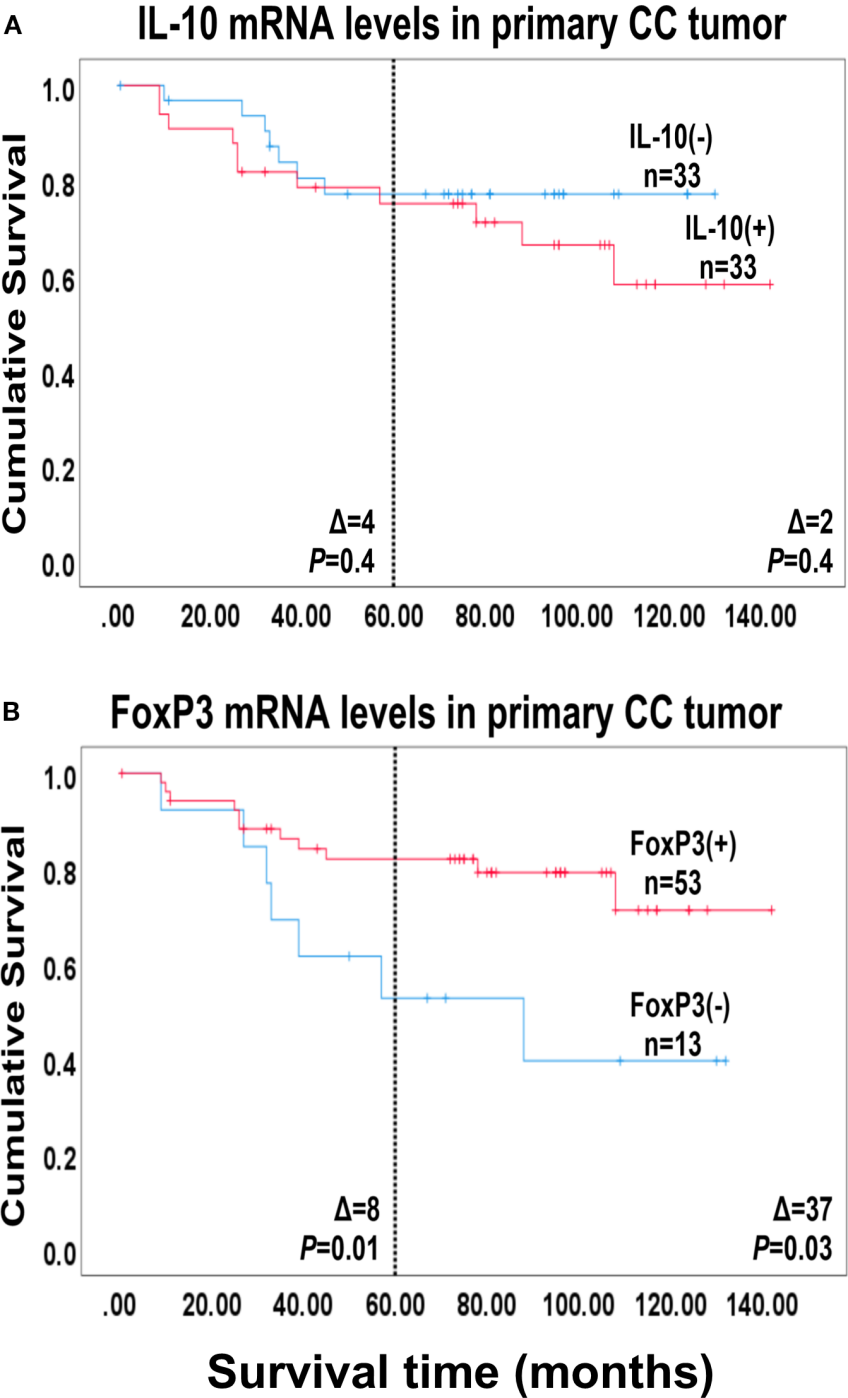
Survival analysis based on IL-10 and FoxP3 mRNA levels in the primary tumor. Kaplan-Meier cumulative survival curves for 66 colon cancer patients divided into two groups, in **(A)** according to the median level of IL-10 mRNA in the primary tumors [0.0014 mRNA copies/18S rRNA unit; IL-10(-) and IL-10(+)] and in **(B)** according to the 20^th^ percentile of FoxP3 mRNA levels of the primary tumors [0.044 FoxP3 mRNA copies/18S rRNA unit; FoxP3(-) and FoxP3(+)]. The patients were followed for 12 years. Differences in disease-free survival time after surgery between the two groups are given as Δ-values in months and statistical significance is given as *p*-values from log-rank test survival analysis. n = number of patients in the respective group.

To investigate whether high levels of IL-10- or FoxP3 mRNA were indicative of good or bad prognosis after surgery we used Cox regression analysis to calculate the hazard ratio for recurrence and Kaplan-Meier survival model combined with the log-rank test to evaluate differences in disease-free survival time after surgery. The results are shown in **Table 2** and **Figures 3A, B**. No significant difference in disease-free survival between patients expressing high or low IL-10 mRNA levels in their primary tumors was seen. In contrast, high levels of FoxP3 mRNA were a sign of good prognosis with a difference in disease-free survival of 8 months at 5-year follow-up after surgery (*p* = 0.01) with a hazard ratio of 0.32. The 20^th^ percentile (0.044 mRNA copies/18S rRNA unit) was used as cut-off for FoxP3.

**Table 2:** Comparative analysis of average survival time and risk for recurrence of disease after surgery of colon cancer (CC) patients with IL-10(-) and IL-10(+) and FoxP3(-) and FoxP3(+) primary tumors.

Patient group	Category	Number of patients in each TNM stage group				Total	5-year follow-up after surgery					12-year follow-up after surgery				
		Stage I	Stage II	Stage III	Stage IV		Disease-free survival ^a^			Risk for recurrence ^b^		Disease-free survival ^a^			Risk for recurrence ^b^	
							Average (Months)	Difference (Months)	*P*-value	Hazard ratio (95% CI)	*P*-value	Average (Months)	Difference (Months)	*P*-value	Hazard ratio (95% CI)	*P*-value
All CC Patients	IL-10(-) ^c^	6	16	8	3	33	53	4	0.4	1.6	0.4	107	2	0.4	1.5	0.4
	IL-10(+)	7	15	9	2	33	49			(0.6-4.1)		105			(0.6-3.9)	
	FoxP3(-) ^d^	1	5	6	1	13	45	8	0.01	0.32	0.02	79	37	0.03	0.35	0.03
	FoxP3(+)	12	26	11	4	53	53			(0.12-0.84)		116			(0.14-0.93)	

a Mean survival time after surgery of CC patients as calculated by cumulative survival analysis according to Kaplan-Meier.

b Hazard ratio, with 95% confidence interval (CI), for risk of recurrence of CC patients as calculated according to univariate COX regression analysis.

c CC patients divided into the two groups IL-10(-) and IL-10(+) using the median value of IL-10 mRNA levels of primary tumors (0.002 mRNA copies/18S rRNA unit) as cut-off.

d CC patients divided into the two groups FoxP3(-) and FoxP3(+) using the 20^th^ percentile of FoxP3 mRNA levels in all CC patients’ primary tumors (0.044 mRNA copies/18S rRNA unit) as cut-off.

### 3.2 Analysis of lymph nodes of CC patients

To investigate whether expression levels in LNs would reflect those in the primary tumor we performed pairwise comparisons using the LN expressing the highest level to compare with the patient's primary tumor (**Table 3**). There was no statistically significant correlation between the expression levels of either IL-10 or FoxP3 mRNAs at the two sites.

**Table 3:** Pairwise comparisons of levels in the primary tumor and the highest lymph node of IL-10 and FoxP3 mRNAs in colon cancer (CC) patients.

Patient group	Pairwise comparison of mRNA levels in the highest lymph node and the corresponding primary tumor			
	IL-10		FoxP3	
	r	*p*-value	r	*p*-value
All CC patients (n=66)	0.006	0.9	-0.1	0.4
Stage I patients (n=14)	0.4	0.1	-0.1	0.7
Stage II patients (n=30)	-0.03	0.9	-0.1	0.5
Stage III patients (n=17)	0.1	0.6	0.05	0.8
Stage IV patients (n=5)	0.3	0.6	-0.7	0.3

The correlation coefficients (r) and the *p*-values were calculated by two-tailed Spearman’s rank order correlation test.

**Figure 4:**
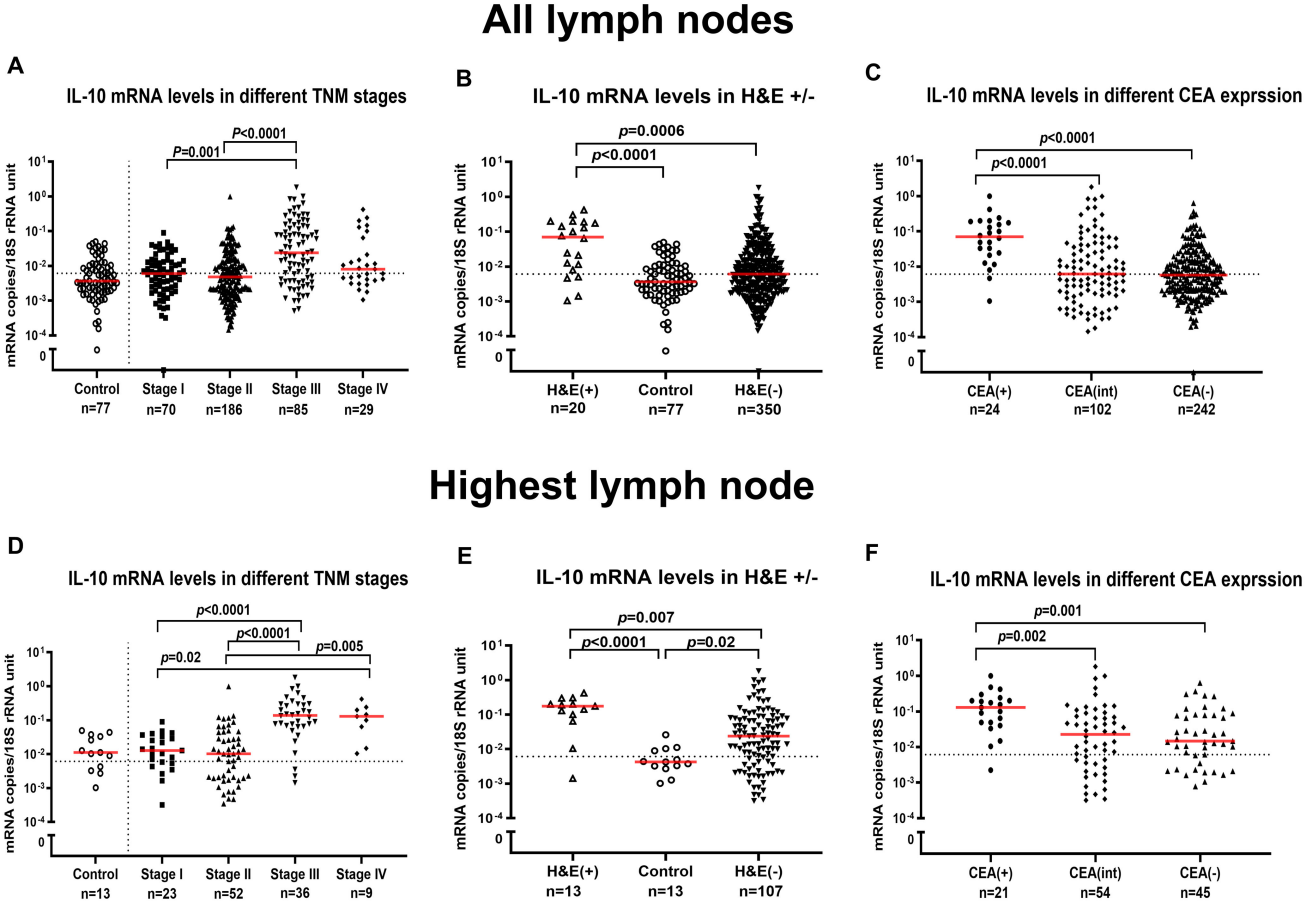
IL-10 mRNA levels in lymph nodes. IL-10 mRNA levels in all lymph nodes **(A-C)** and in the lymph node with the highest level for each patient **(D-F)**. **(A, D)** show IL-10 mRNA levels in lymph nodes of non-cancerous disease patients (Control) and colon cancer patients in different TNM stage (Stage I–IV). **(B, E)** show IL-10 mRNA levels in metastatic lymph nodes of colon cancer patients [H&E(+)], non-metastatic nodes of colon cancer patients [H&E(-)] and lymph nodes of non-cancerous disease patients (Control). **(C, F)** show IL-10 mRNA levels in lymph nodes categorized into three groups according to their CEA mRNA levels [CEA(-) = CEA mRNA levels <0.013 copies/18S rRNA unit, CEA(int) = intermediate CEA mRNA levels, that is 0.013 to 3.67 copies/18S rRNA unit, and CEA(+) = CEA mRNA levels >3.67 copies/18S rRNA unit]. Dashed horizontal lines indicate the clinical cutoff value of IL-10 mRNA (0.006 mRNA copies/18S rRNA unit). Red horizontal lines indicate median values. n = number of analyzed lymph node samples. *p*-values were calculated by Kruskal–Wallis non-parametric ANOVA, followed by *post hoc* Dunn’s test for multiple comparisons.

The mRNA levels of IL-10 and FoxP3 in 370 LNs from 120 CC patients representing all four TNM stages and in 77 LNs of 13 control patients were determined. The results are shown in **Figure 4** and in **Supplementary Figure S1**. The results are shown both for all LNs and for the LN with the highest value of the patient. For IL-10 mRNA there was a highly significant difference between stage III patients on one hand and stage I and II patients on the other. If only the highest LN was analyzed, stage IV LNs also expressed significantly higher levels than the two low TNM stages (**Figures 4A, D**). Significantly higher IL-10 mRNA levels were also seen in H&E(+) LNs compared with both H&E(-) LNs and control LNs (**Figures 4B, E**). Finally, if CEA(+) LNs were compared with CEA(-) nodes or nodes with intermediate CEA levels, the former expressed significantly higher IL-10 mRNA levels (**Figures 4C, F**). We conclude, that IL-10 mRNA levels in LNs are positively correlated with the presence of tumor cells both as defined by histopathology and by the tumor marker CEA. In contrast to IL-10 mRNA FoxP3 mRNA expression in LNs did not show any variation relative to either TNM stage, H&E positivity, or to CEA mRNA expression level (**Supplementary Figure S1**).

To investigate whether the positive results obtained for IL-10 mRNA determinations in LNs of CC patients was in some way dependent on the house-keeping gene, i.e. 18S rRNA, we also calculated the IL-10 mRNA : FoxP3 mRNA ratio in relation to TNM stage, H&E positivity, and CEA mRNA level (**Supplementary Figure S2**). The result was identical to that obtained by determining IL-10 mRNA copies/18S rRNA unit supporting the conclusion that IL-10 mRNA expression levels are correlated to presence of tumor cells in LNs.

**Figure 5:**
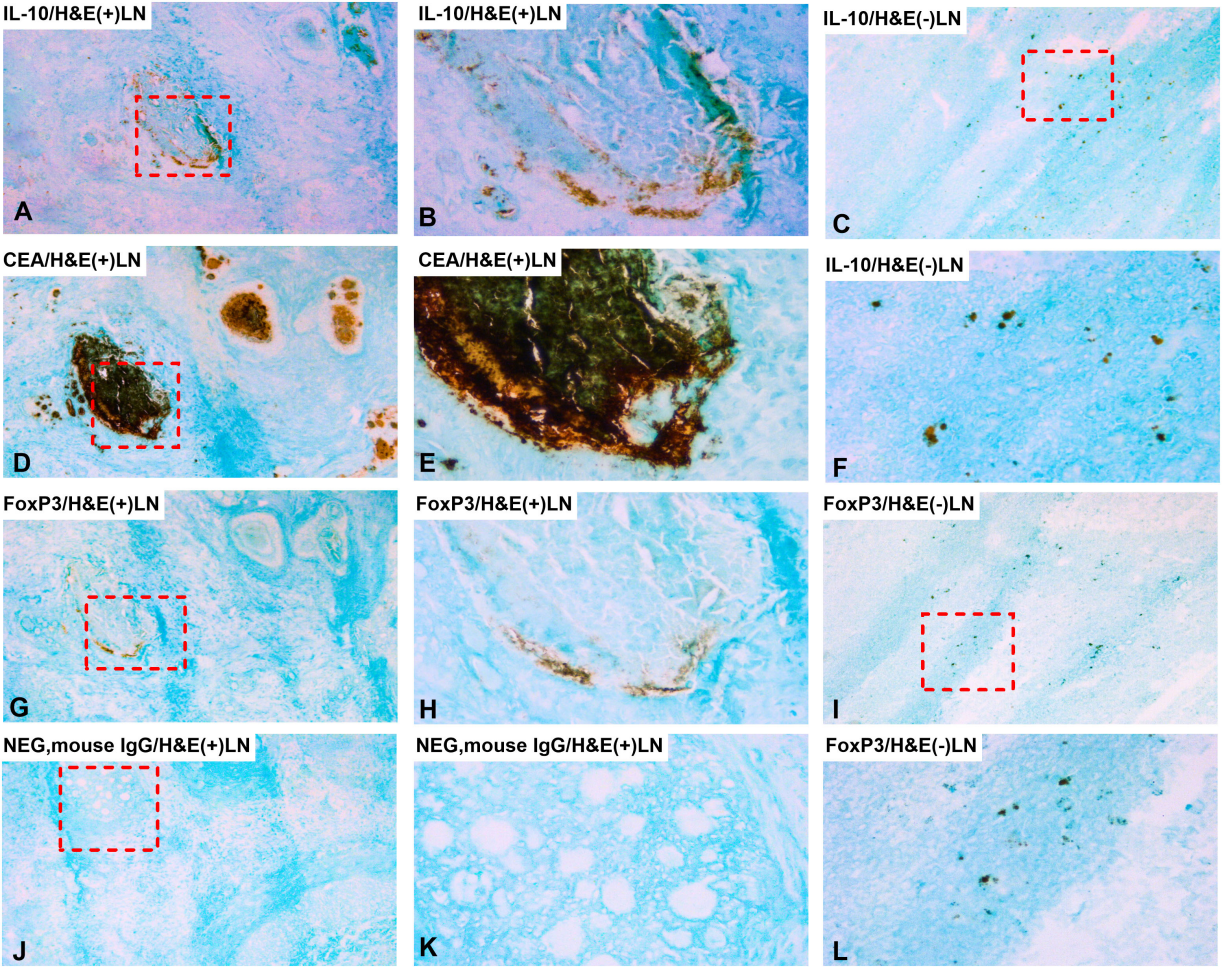
Distribution of IL-10 and FoxP3 positive cells in metastatic and non-metastatic lymph nodes. Immunoperoxidase stained tissue sections of lymph nodes of a colon cancer patient. **(A, B, D, E, G, H, J, K)** show sections of a metastatic lymph node [H&E(+)] and **(C, F, I, L)** a non-metastatic lymph node [H&E(-)]. **(A)** H&E(+) lymph node stained with anti-IL-10 mAb in a consecutive section of **(D)**, original magnification 100×. **(B)** higher magnification of the area indicated by a hatched box in **(A)**, 400x. **(C)** H&E(-) lymph node stained with anti-IL-10 mAb, original magnification 100x. **(D)** H&E(+) lymph node stained with anti-CEA mAb, original magnification 100×. **(E)** higher magnification of the area indicated by a hatched box in **(D)**, 400×. **(F)** higher magnification of the area indicated by a hatched box in **(C)**, 400×. **(G)** H&E(+) lymph node stained with anti-FoxP3 mAb in a consecutive section of **(D)**, original magnification, 100×. **(H)** higher magnification of the area indicated by a hatched box in **(G)**, 400x. **(I)** H&E(-) lymph node stained with anti-FoxP3 mAb, original magnification,100x. **(J)** negative control (mouse IgG) of an H&E(+) lymph node, original magnification 100×. **(K)** higher magnification of the area indicated by a hatched box in **(J)**, 400×. **(L)** higher magnification of the area indicated by a hatched box in **(I)**, 400×. **(A, D, G)** are consecutive sections.

IL-10 and FoxP3 expressions at the protein level were determined also in LNs by using the consecutive immunohistochemistry technique. **Figure 5** shows a representative result. Anti-CEA mAb identified several tumor cell aggregates in the LN (**Figures 5D, E**). In H&E(+) LNs, both anti-IL-10 and anti-FoxP3 mAbs detect cells in close vicinity to the CEA positive tumor cell aggregates (**Figures 5A, B** and **Figures 5G, H**) strongly supporting the view that cells in the tumor cell microenvironment express both biomarkers. Occasionally a few IL-10 and FoxP3 positive cells were seen in H&E(-) LNs (**Figures 5C, F** and **Figures 5I, L**).

**Figure 6:**
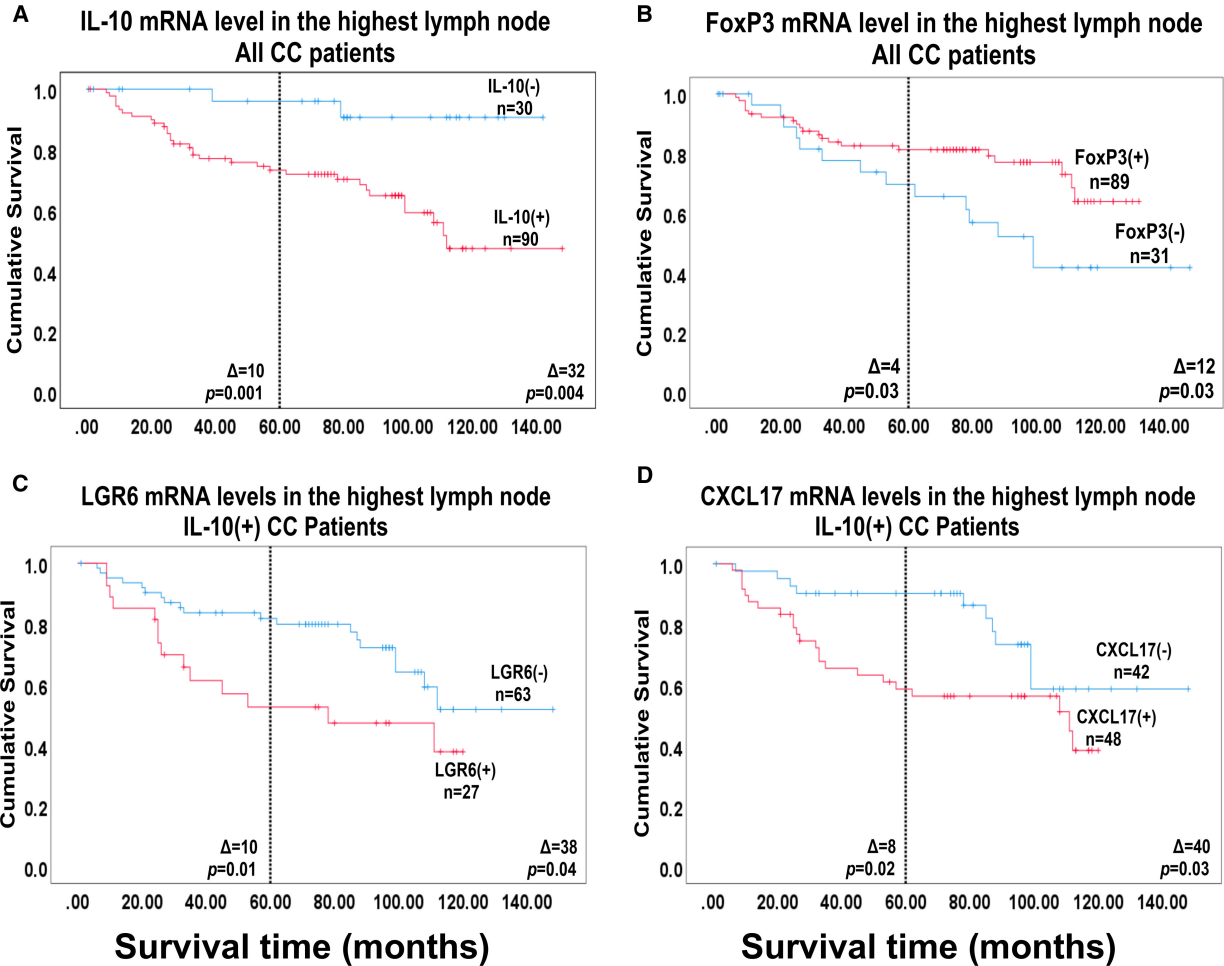
Survival analysis based on IL-10 mRNA levels in lymph nodes. Kaplan-Meier cumulative survival curves of colon cancer patients divided into two groups according to biomarker mRNA levels. **(A)** all 120 colon cancer patients divided into IL-10(+) and IL-10(-) groups using the median value of IL-10 mRNA levels in lymph nodes of patients in the H&E(-) group (0.006 mRNA copies/18S rRNA unit) as cutoff. **(B)** all 120 colon cancer patients divided into FoxP3(+) and FoxP3(-) groups using the median value of FoxP3 mRNA levels in lymph nodes in the H&E(+) group (1.016 mRNA copies/18S rRNA unit) as cutoff. **(C)** 90 colon cancer patients with IL-10 mRNA levels >0.006 copies/18S rRNA unit in their highest lymph node [IL-10(+)] divided into LGR6(+) and LGR6(-) groups using the established cutoff 0.0471 LGR6 mRNA copies/18S rRNA unit (reference 29). **(D)** 90 IL-10(+) colon cancer patients divided into CXCL17(+) and CXCL17(-) groups using the established cutoff >0.0003 CXCL17 mRNA copies/18S rRNA unit (reference 28). The dashed vertical lines indicate 5-year follow-up after surgery. Differences in disease-free survival time after surgery between the two groups are given as Δ-values in months and statistical significance as *p*-values from log-rank test of survival analysis.

The utility of IL-10 mRNA analysis of LNs either alone or in combination with other biomarkers for predicting outcome was investigated. **Figure 6** and **Table 4** summarize the results. Of greatest interest are the findings with IL-10 alone and IL-10 in combination with LGR6. IL-10 alone separates the patients into two groups one with very low risk of recurrence expressing low levels of IL-10 and another group with significantly worse risk expressing high IL-10 mRNA levels (**Figure 6A**: Δ = 10 months at 5 years, *p* = 0.001; **Table 4**: hazard ratio = 12.4 at 5-year follow-up, *p* = 0.01). Further division of the IL-10 high group is achieved both with LGR6 and CXCL17 (**Figures 6C, D**). Note that the group with high levels of the marker is having the worst prognosis. For LGR6 the group with high levels contained 27 patients (Δ = 10 months at 5-year of follow-up, *p* = 0.01; **Table 4**: hazard ratio = 2.4 at 5 years, *p* = 0.02). Thus, by combining the two markers, three groups of patients with clearly different prognosis from good, to relatively poor to very poor can be identified. Using CXCL17 as the partner to IL-10, a similar division into three risk groups was achieved (**Figure 6D**).

**Table 4:** Comparative analysis of average survival time after surgery and risk for recurrence of disease of colon cancer (CC) patients with IL-10(-) and IL-10(+) and FoxP3(-) and FoxP3(+) lymph nodes and patients with IL10(+) lymph nodes combined with other biomarkers.

Patient group	Category	Number of patients in each TNM stage group				Total	5-year follow-up after surgery					12- year follow-up after surgery				
		Stage I	Stage II	Stage III	Stage IV		Disease-free survival^a^			Risk for recurrence^b^		Disease-free survival^a^			Risk for recurrence^b^	
							Average (Months)	Difference (Months)	*P*- value	Hazard ratio (95% CI)	*P*-value	Average (Months)	Difference (Months)	*P*-value	Hazard ratio (95% CI)	*P*- value
All CC Patients	IL-10(-)^c^	7	20	3	0	30	59	10	0.001	12.4	0.01	134	32	0.004	6.3	0.01
	IL-10(+)	16	32	33	9	90	49			(1.7-91.2)		102			(1.5-26.3)	
	FoxP3(-)^d^	5	11	12	3	31	48	4	0.03	0.5	0.04	94	12	0.03	0.5	0.03
	FoxP3(+)	18	41	24	6	89	52			(0.23-0.96)		106			(0.24-0.95)	
IL10(+) CC patients^e^	CXCL17(-)^f^	9	17	15	1	42	53	8	0.02	2.5	0.02	117	40	0.03	2.2	0.04
	CXCL17(+)	7	15	18	8	48	45			(1.2-5.4)		77			(1.0-4.6)	
	LGR6(-)^g^	13	25	23	2	63	52	10	0.01	2.4	0.02	110	38	0.04	2.1	0.04
	LGR6(+)	3	7	10	7	27	42			(1.2-4.8)		72			(1.0-4.1)	

a Mean survival time after surgery for CC patients as calculated by cumulative survival analysis according to Kaplan–Meier.

b Hazard ratio, with 95% confidence interval (CI), for risk of recurrence of CC patients as calculated according to univariate COX regression analysis.

c CC patients divided into the two groups IL-10(-) and IL-10(+) using the median value of IL-10 mRNA levels in lymph nodes from CC patients in the H&E(-) group (0.006 mRNA copies/18S rRNA unit) as cut-off.

d CC patients divided into the two groups FoxP3(-) and FoxP3(+) using the median value of FoxP3 mRNA levels in lymph nodes from CC patients in the H&E(+) group (1.016 mRNA copies/18S rRNA unit) as cut-off.

e CC patient group with IL-10 mRNA levels above 0.006 mRNA copies/18S rRNA unit.

f CC patients divided into the two groups CXCL17(-) and CXCL17(+) using a cut-off value of 0.0003 mRNA copies/18S rRNA unit (ref. 28).

g CC patients divided into the two groups LGR6(-) and LGR6(+) using a cut-off value of 0.0471 mRNA copies/18S rRNA unit (ref. 29).

Dividing LNs into a FoxP3 mRNA high and a low expression group demonstrated, firstly, that the prognosis is worse for the group with low FoxP3 mRNA levels and, secondly, the difference is small and probably not clinically useful (**Figure 6B**).

**Supplementary Table S1** shows the results of correlation analysis between IL-10 mRNA levels and FoxP3-, CXCL17- and LGR6 mRNA levels in LNs of CC patients. Compatible with the survival data, the best correlation was seen between IL-10 and LGR6, followed by IL-10 and CXCL17 while there was poor correlation between IL-10 and FoxP3.

## 4 Discussion

This study is compatible with the notion that IL-10 expressing cells in the regional LNs of CC patients has a tumor growth promoting role. Thus, high levels of IL-10 mRNA in LNs of CC patients indicate poor survival after surgery in patients operated for cure. Reversely, low levels of IL-10 mRNA indicate a very low risk of recurrence up to 12 years of follow-up. The tumor promoting role of cells expressing high levels of IL-10 mRNA is not seen in the primary tumor. Whether this points to a dual role of IL-10 – inhibiting or promoting tumor growth depending on the susceptibility of the tumor cells at different stages of their development or due to interaction between IL-10 producing cells and other cells in the two environments is an open question.

In line with our finding that IL-10 mRNA levels are significantly higher in LNs from TNM stage III patients compared to the primary site (12 times concentration difference) is the work by Townsend et al. (15). These authors used an immunohistochemical assay to quantify IL-10 protein levels in entire tissue sections comparing primary tumor with metastatic sites. An elevated expression level of IL-10 in the metastatic site was found while a parallel analysis of TGF-β did not show a difference between the two sites. Moreover, high levels of IL-10 in regional LNs were found to be associated with metastasis (32).

Our immunohistochemical results indicate that the IL-10 producing cells belong to cells of the tumor microenvironment at both the primary and secondary sites and that increase in IL-10 levels is not due to ectopic expression in the tumor cells themselves. Support for this notion is that none of 5 CC cell lines investigated here expressed IL-10 mRNA, a result in line with the findings by Uhlén et al., in Human Protein Atlas (33), where 46 of 48 human CC cell lines were IL-10 mRNA negative. It is likely that the IL-10 producing cells in the LNs of CC patients are regulatory T-cells since a closely similar staining pattern was obtained with mAbs against FoxP3. Our finding in LNs of CC patients is in line with a recent study by Shiri et al., 2024 (34) who demonstrated, in animal models of colon cancer, that IL-10 produced by regulatory T-cells activate monocytes locally to upregulate PD-L1 which in turn interacts with PD1 on cytotoxic T-cells (CTLs) to inhibit their production of granzyme B. This inhibition of cytotoxicity by the CTLs promoted growth of liver metastases. Further studies are needed to reveal the mechanism behind the tumor promoting effect of IL-10 at secondary sites in humans.

The second take home lesson from this study is that selected biomarkers can successfully be combined with IL-10 to achieve further subdivision of the IL-10(+) patients into groups with different degrees of risk of recurrence. Here we show that combination of IL-10 with LGR6 allows further division of the IL-10(+) group. Thus, these two biomarkers give rise to three risk groups - very low risk, high risk and very high risk requiring different treatment strategies. Similar results were obtained by combining IL-10 with CXCL17. CXCL17 was chosen from our previous studies on the capacity of cytokines/chemokines to predict recurrence in CRC. In all we have investigated eight pleiotropic chemokines belonging to different ligand – receptor families with the potential to influence the micromilieu of the tumor in different ways. These were CCL2, CXCL9, CXCL10, CXCL12, CXCL14, CXCL16, CXCL17 and MIF (24, 25, 28, 31, 35). Of these, CXCL16 and CXCL17 had the highest prognostic value when expressed at high levels in LNs. Interestingly, both were ectopically expressed in the tumor cells.

FoxP3 mRNA analysis has little value as a prognostic marker in CC. Although there was a difference in levels in the primary tumor of patients with poor prognosis compared to those with better prognosis, the difference was small. Moreover, FoxP3 levels in patient's regional LNs did not vary with TNM-stage or CEA mRNA levels. The result is perhaps surprising since FoxP3 is considered to be a marker for regulatory T cells, but FoxP3 was also found to be expressed in other cells including fibroblasts.

In our studies on the prognostic value of biomarkers we have focused on mRNA analyses of tumor tissue using real-time qRT-PCR with RNA copy standard because we consider this method to be very suitable for a clinical setting. The method is highly sensitive and specific, allows quantification of different or all splice variants of a biomarker by appropriate choice of primers and probe, requires very limited amount of RNA, is robust and easily adaptable for automation. Furthermore, several biomarkers can be analyzed in parallel in the same aliquot of a sample as we demonstrated in the COLONODE-study in which 20 LN samples were analyzed for levels of 5 biomarker mRNAs and a house-keeping gene in one qRT-PCR run (36). In our view this method is superior to RNA-seq or analyses at the protein level, such as Western-blot or ELISA, for prognostic evaluation because of the higher specificity and sensitivity as well as the smaller amount of tissue required. A disadvantage is, however, that analyses at the mRNA level do not allow investigation of posttranslational modifications of the biomarker protein. Microscopic examination of primary and secondary tumor tissue has lower prognostic value, even when using specific monoclonal antibodies in immunohistochemistry (27, 36, 37). These methods provide spatial distribution and localization within the tumor tissue and are therefore useful for understanding of mechanisms of interaction in the tumor microenvironment.

For liquid biopsies, including vesicles in blood, plasma, serum and urine, analysis at the protein level is probably preferrable over mRNA analysis because no good method for normalization of mRNA levels is yet available. On the other hand, the capacity of a biomarker to predict outcome might be blurred by the systemic immune status of the patient giving different background levels of cytokines as IL-10.

A limitation with this study is the relatively small number of CC patients that was included - 120 patients donating 370 LNs. Moreover, the majority of patients were of Scandinavian origin. Future prospective studies with comprehensive clinical documentation are warranted to validate and expand our findings.

## 5 Conclusion

Determination of IL-10 mRNA levels in regional LNs is useful for prediction of outcome for CC patients receiving curative surgery. Low levels of IL-10 mRNA is an excellent marker to identify those patients who do not need further treatment after surgery, while IL-10 in combination with LGR6 or CXCL17 can be used for identification of patients at very high risk of recurrence.

## Data Availability

The original contributions presented in the study are included in the article/**Supplementary Material**. Further inquiries can be directed to the corresponding author.

## Supplementary material

Supplementary Figure 1 FoxP3 mRNA levels in lymph nodes. FoxP3 mRNA levels in all lymph nodes **(A-C)** and the lymph node with the highest level for each patient **(D-F)**. **(A, D)** show FoxP3 mRNA levels in lymph nodes of non-cancerous disease patients (Control) and colon cancer patients in different TNM stage (Stage I–IV). **(B, E)** show FoxP3 mRNA levels in metastatic lymph nodes of colon cancer patients [H&E(+)],non-metastatic nodes of colon cancer patients [H&E(-)] and lymph nodes of non-cancerous disease patients (Control). **(C, F)** show FoxP3 mRNA levels in lymph nodes categorized into three groups according to their CEA mRNA levels [CEA(-) = CEA mRNA levels <0.013 copies/18S rRNA unit, CEA(int) = intermediate CEA mRNA levels, that is 0.013 to 3.67 copies/18S rRNA unit, and CEA(+) = CEA mRNA levels >3.67 copies/18S rRNA unit]. Dashed horizontal lines indicate the clinical cutoff value of FoxP3 mRNA (1.016 mRNA copies/18S rRNA unit). Red horizontal lines indicate median values. n = number of analyzed lymph node samples. *p*-values were calculated by using Kruskal–Wallis non-parametric ANOVA, followed by *post hoc* Dunn’s test for multiple comparisons.

Supplementary Figure 2 IL-10:FoxP3 mRNA ratio in lymph nodes.IL-10:FoxP3 ratio in lymph nodes of 120 colon cancer patients and 13 patients with non-cancerous disease. Each patient is represented by the lymph node with the highest ratio. **(A)** IL-10:FoxP3 ratio in lymph nodes from non-cancerous disease patients (Control) and colon cancer patients in different TNM stage (Stage I–IV). **(B)** IL-10:FoxP3 ratio in metastatic lymph nodes of colon cancer patients [H&E(+)],non-metastatic nodes of colon cancer patients [H&E(-)], and lymph nodes of non-cancerous disease patients (Control). **(C)** shows IL-10:FoxP3 ratio in lymph nodes categorized into three groups according to their CEA mRNA levels [CEA(-) = CEA mRNA levels <0.013 copies/18S rRNA unit, CEA(int) = intermediate CEA mRNA levels, that is 0.013 to 3.67 copies/18S rRNA unit, and CEA(+) = CEA mRNA levels >3.67 copies/18S rRNA unit]. The ratio was calculated by dividing the IL-10 mRNA level by the FoxP3 mRNA level of the same lymph node. n = number of lymph node samples, one per patient. Red horizontal lines indicate the median values. Dashed horizontal lines indicate the clinical cutoff value (0.005). *p*-values were calculated by using Kruskal–Wallis non-parametric ANOVA, followed by *post hoc* Dunn’s test for multiple comparisons.
