# Supplementary figures and images for: High IL-10 mRNA levels in regional lymph nodes of colon cancer patients indicate poor prognosis
Source: Front Immunol. 2025 Sep 23;16:1589533. doi: 10.3389/fimmu.2025.1589533 (PMC12500745; doi:10.3389/fimmu.2025.1589533)

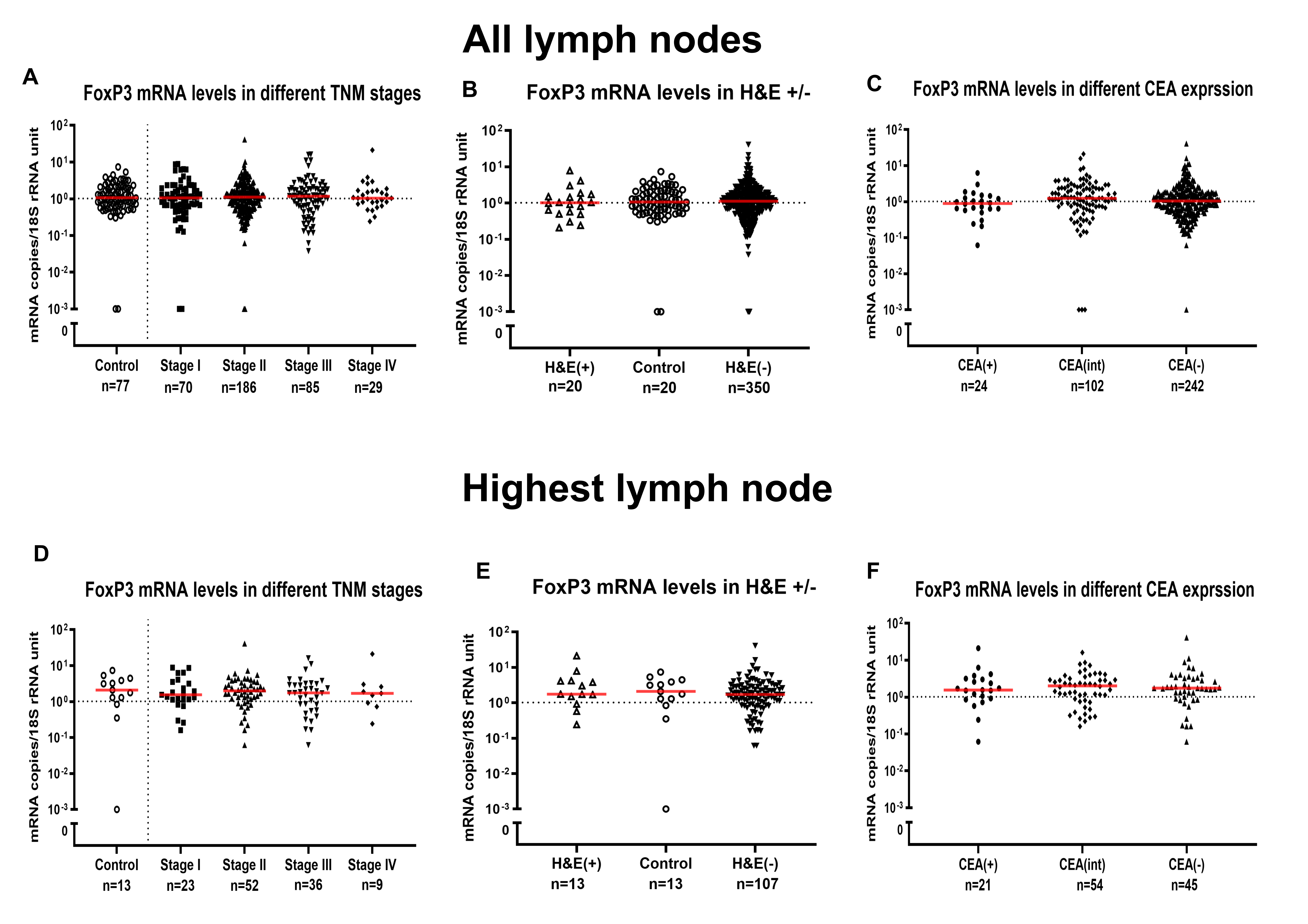

Supplement: Supplementary Figure 1 — FoxP3 mRNA levels in lymph nodes. FoxP3 mRNA levels in all lymph nodes (A-C) and the lymph node with the highest level for each patient (D-F). (A, D) show FoxP3 mRNA levels in lymph nodes of non-cancerous disease patients (Control) and colon cancer patients in different TNM stage (Stage I–IV). (B, E) show FoxP3 mRNA levels in metastatic lymph nodes of colon cancer patients [H&E(+)],non-metastatic nodes of colon cancer patients [H&E(-)] and lymph nodes of non-cancerous disease patients (Control). (C, F) show FoxP3 mRNA levels in lymph nodes categorized into three groups according to their CEA mRNA levels [CEA(-) = CEA mRNA levels <0.013 copies/18S rRNA unit, CEA(int) = intermediate CEA mRNA levels, that is 0.013 to 3.67 copies/18S rRNA unit, and CEA(+) = CEA mRNA levels >3.67 copies/18S rRNA unit]. Dashed horizontal lines indicate the clinical cutoff value of FoxP3 mRNA (1.016 mRNA copies/18S rRNA unit). Red horizontal lines indicate median values. n = number of analyzed lymph node samples. p-values were calculated by using Kruskal–Wallis non-parametric ANOVA, followed by post hoc Dunn’s test for multiple comparisons. [file Image1.tif]

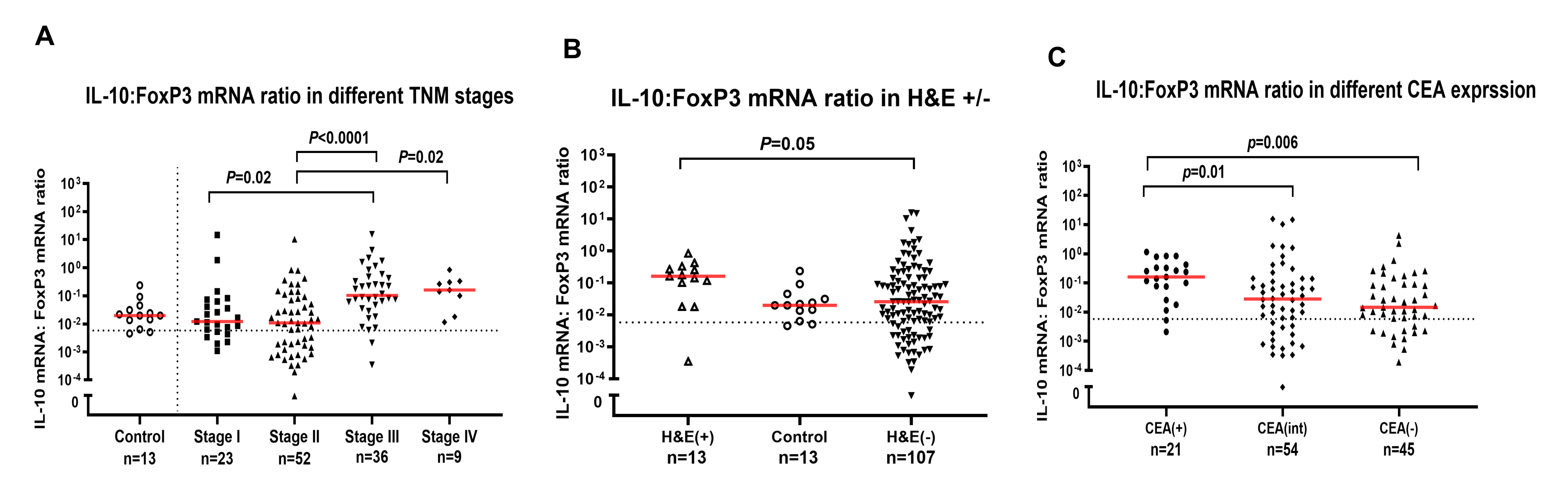

Supplement: Supplementary Figure 2 — IL-10:FoxP3 mRNA ratio in lymph nodes.IL-10:FoxP3 ratio in lymph nodes of 120 colon cancer patients and 13 patients with non-cancerous disease. Each patient is represented by the lymph node with the highest ratio. (A) IL-10:FoxP3 ratio in lymph nodes from non-cancerous disease patients (Control) and colon cancer patients in different TNM stage (Stage I–IV). (B) IL-10:FoxP3 ratio in metastatic lymph nodes of colon cancer patients [H&E(+)],non-metastatic nodes of colon cancer patients [H&E(-)], and lymph nodes of non-cancerous disease patients (Control). (C) shows IL-10:FoxP3 ratio in lymph nodes categorized into three groups according to their CEA mRNA levels [CEA(-) = CEA mRNA levels <0.013 copies/18S rRNA unit, CEA(int) = intermediate CEA mRNA levels, that is 0.013 to 3.67 copies/18S rRNA unit, and CEA(+) = CEA mRNA levels >3.67 copies/18S rRNA unit]. The ratio was calculated by dividing the IL-10 mRNA level by the FoxP3 mRNA level of the same lymph node. n = number of lymph node samples, one per patient. Red horizontal lines indicate the median values. Dashed horizontal lines indicate the clinical cutoff value (0.005). p-values were calculated by using Kruskal–Wallis non-parametric ANOVA, followed by post hoc Dunn’s test for multiple comparisons. [file Image2.tif]
