# Supplementary material for: High IL-10 mRNA levels in regional lymph nodes of colon cancer patients indicate poor prognosis
Source: Front Immunol. 2025 Sep 23;16:1589533. doi: 10.3389/fimmu.2025.1589533 (PMC12500745; doi:10.3389/fimmu.2025.1589533)
Supplement: Supplementary file 3 [file Table1.docx]

**Supplementary Table S1.** Correlations between mRNA expression levels of IL-10 and different biomarkers in lymph nodes of colon cancer patients.

|  | | **FoxP3** | | **CXCL17** | | **LGR6** | |
| --- | --- | --- | --- | --- | --- | --- | --- |
|  |  | **r** | ***p*-value** | **r** | **p-value** | **r** | ***p*-value** |
| **IL-10** | **All CC LNs** | -0.1 | 0.04 | 0.2 | 0.001 | 0.4 | <0.0001 |
|  | **TNM Stage I LNs** | 0.2 | 0.06 | 0.4 | 0.0007 | 0.5 | <0.0001 |
|  | **TNM Stage II LNs** | -0.3 | 0.0006 | -0.1 | 0.1 | 0.5 | <0.0001 |
|  | **TNM Stage III LNs** | -0.05 | 0.7 | 0.2 | 0.08 | 0.2 | 0.03 |
|  | **TNM Stage IV LNs** | -0.3 | 0.2 | 0.5 | 0.01 | 0.6 | 0.0002 |

The correlation coefficients (r) and the *p*-values were calculated by two-tailed Spearman’s rank order correlation test.

LN = lymph node; CC = colon cancer; TNM = tumor-node-metastasis.
